# Is Losing Weight Worth Losing Your Kidney: Keto Diet Resulting in Renal Failure

**DOI:** 10.7759/cureus.36546

**Published:** 2023-03-22

**Authors:** Girma M Ayele, Rediet Tefera Atalay, Ruth T Mamo, Siham Hussien, Beimnet Nigussie, Abel Fissha, Miriam B Michael

**Affiliations:** 1 Internal Medicine, Howard University Hospital, Washington D.C., USA; 2 General Medicine, Nordic Medical Center, Addis Ababa, ETH; 3 Internal Medicine, University of Maryland Midtown Campus, Baltimore, USA; 4 Pathology, Wolkite University, Wolkite, ETH; 5 Internal Medicine, International Cardiovascular and Medical Center (iCMC) Hospital, Addis Ababa, ETH; 6 Internal Medicine, University of Maryland, Baltimore, USA

**Keywords:** keto diet and acute kidney injury, keto diet, low carbohydrate diet, weight loss, renal failure, ketogenic diet

## Abstract

Keto diet is defined as a high-fat, adequate-protein, and low-carbohydrate nutrition which forces the body to burn fats and use an alternative metabolic fuel resource by stimulating endogenous ketone production. The standard range of ketones in ketosis is up to 3.00mmol/L, and anything beyond this level can result in serious medical conditions. This diet's most common and easily reversible consequences are constipation, low-grade acidosis, hypoglycemia, kidney stones, and increased lipid in the blood. We present a case of a 36-year-old female who presented with pre-renal azotemia after starting a keto diet regimen.

## Introduction

The ketogenic diet has become the most popular diet increasingly for weight loss. It has proved its effectiveness for weight loss in obese individuals and with decreases in hyperlipidemia and cardiovascular risk factors [1]. The diet works by creating a state called “physiologic ketosis" and the exact effect on the body is unclear, making a lot of physicians skeptical about its use. Some complications associated with the ketogenic diet include renal impairment and decreased bone density [1].

Renal dysfunction associated with the ketogenic diet has been reported in individuals with underlying kidney disease exposed to high protein. But, studies on individuals with normal renal function showed no renal impairment [2]. We present a 36-year-old female patient with no underlying renal problem who developed acute renal injury after she started a ketogenic diet.

## Case presentation

This is a 36-year-old female patient who presented with three days duration of persistent nausea and vomiting, along with fatigue and malaise. She stated she started a new weight loss journey with diet and exercise. She was on a ketogenic diet for the past two months and lost more than 30 pounds. Her diet primarily consisted of three meals a day: mainly composed of eggs, beef, avocado, chicken, and peanuts. She had no change in urine output and denied any recent medication intake. She did not have a history of hypertension, diabetes, or kidney disease in the past. She states that her baseline renal function test was normal during routine check up which was done eight months back.

At presentation, vital signs were normal. Physical examination was unremarkable, except for mild signs of dehydration like dry buccal mucosa and mild epigastric tenderness. Laboratory investigation showed elevated creatinine of 4.3 meq/L, hypokalemia of 3.3 meq/L, FeNa was 4% and positive urine ketone. Urine electrolytes, urine microscopy, complete blood count, liver function test, rheumatoid factor, anti-nuclear antibody, hepatitis B, and hepatitis C were all normal. Abdominal ultrasound showed only cholelithiasis. Renal function test trends are shown in Table 1 below.

**Table 1:** Laboratory result BUN: Blood urea nitrogen Cr: Creatinine H: High N: Normal

Date	6/22/2020	6/23/2020	6/24/2020	6/25/2020	6/27/2020	7/4/20	8/5/20
Creatinine	4.53 (H)	4 (H)	3.55 (H)	2.85 (H)	1.83 (H)	0.82 (N)	0.84 (N)
BUN	52 (H)	42 (H)	47 (H)	42 (H)	27 (H)	20 (N)	18 (N)
Urine Cr	0.25 (N)	-	-	-	-	-	-
Urine protein	5.1 (N)	-	-	-	-	-	-

She was given the diagnosis of acute kidney injury induced by a ketogenic diet and cholelithiasis due to weight loss. She was given IV fluids and supportive care. She showed improvement in symptoms by the second day, and her creatinine level started to drop. She was discharged on the fifth day of admission with normal creatinine.

## Discussion

A ketogenic diet comprises mostly fats, some protein, and very few carbohydrates. The breakdown of macronutrients is typically 60% fat, 35% protein, and 10% carbohydrates. In a daily diet of 2000 calories, carbohydrates usually range from 20 to 50 grams. The term "ketogenic diet." was created by Russell Wilder in 1921 and was initially utilized to treat epilepsy. It was popular for about 10 years as a treatment for pediatric epilepsy but lost favor when antiepileptic drugs came on the market. The idea of the ketogenic diet making a comeback as a quick weight-loss strategy is very recent [3].

Our body uses carbohydrates as a primary energy source. When carbohydrate intake drops below 50 grams per day, insulin secretion decreases dramatically, and the body enters a state of breakdown. Various metabolic adjustments are compelled as the body's glycogen reserves are depleted. When the body's tissues are deficient in carbohydrates, two metabolic processes, gluconeogenesis and ketogenesis, come into play [4,5]. Gluconeogenesis is the process by which the body produces glucose internally. If glucose levels in the body become too low, the body's natural glucose production cannot meet its energy needs, leading to the start of ketogenesis, which provides energy in the form of ketone bodies. The primary source of energy is switched to ketone. During ketosis, glucose and fat storage will decrease due to decreased insulin secretion. Other changes during ketosis, including hormonal changes, may also facilitate fat degradation. Acetoacetate, produced during the metabolism of fatty acids, is then changed into acetone and beta-hydroxybutyrate. Nutritional ketosis is the term used to refer to the state of the body where there is an increased level of ketone bodies. The body's metabolism remains in a state of ketosis for as long as it is denied carbohydrates. Ketone bodies are generated in low amounts without changing the blood's pH level. This is different from ketoacidosis, which is a dangerous situation where there is a significant increase in ketone body production, causing an acidic shift in the blood's pH [3].

**Figure 1:**
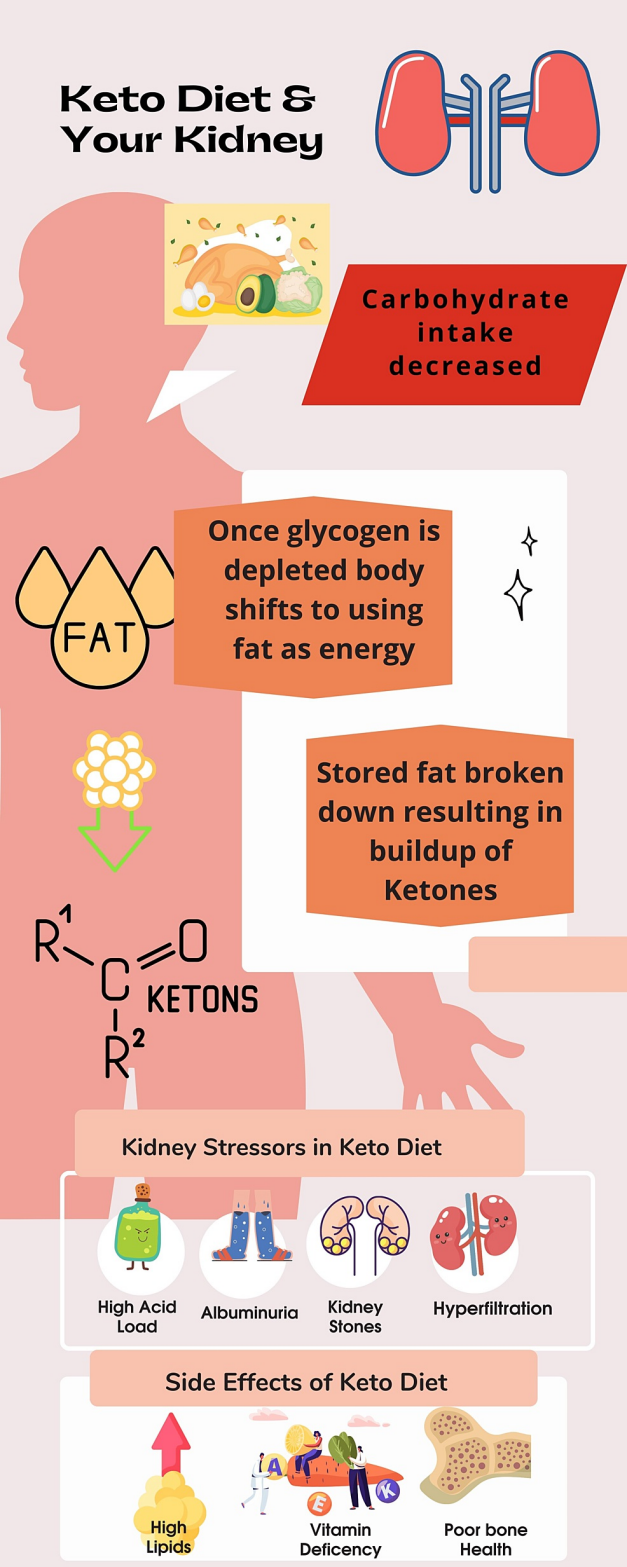
Pathophysiology of the keto diet and its complications. Image created by Dr. Miriam Michael

The ketogenic diet is becoming more popular as a weight loss regimen; however, the evidence has failed to show clinically significant benefits over comparator diets. Even worse, it may cause hyperlipidemia, vitamin and mineral deficiencies, fatigue, and kidney damage, among other complications (Figure 1) [6]. Increased acid production from a ketogenic diet may cause metabolic acidosis and related issues, including weakened bone health [2]. Studies show that people with chronic kidney disease and those without prior kidney problems are experiencing a decline in kidney function. The average required protein intake for individuals with ideal body weight is 0.6 grams per kilogram. There is no exact definition for a high-protein diet, but most definitions consider a range of 1.2 to 2.0 grams of protein per kilogram of ideal body weight. Typically, a protein intake of over 1.5 grams per kilogram of ideal body weight is considered a high-protein diet [2]. The proposed mechanisms of high protein dietary intake for kidney damage include mediators including acid load, high phosphate content, gut microbiome dysbiosis, and inflammation. High protein intake has been shown to directly cause an increase in kidney volume and weight in a mouse model, resulting in the expansion of the mesangial matrix and tubulointerstitial fibrosis [2]. In experimental rat studies, it has been suggested that increasing the protein dose increased the expression of proinflammatory genes. In pig models, consuming a high-protein diet over a long period resulted in 55% more renal fibrosis and 30% more glomerulosclerosis [2]. Additionally, it is known that consuming animal protein leads to a higher incidence of kidney stones. This has been observed in the pediatric epilepsy community, where a ketogenic diet was used as a treatment for epilepsy in children, and nephrolithiasis was listed as one of the side effects [7].

Studies have shown that the source of the dietary protein also matters; it is noted that animal protein has a higher incidence of kidney injury compared to plant-based protein [8]. One of the largest human trials showed that a high protein diet increased estimated glomerular filtration (eGFR) by 3.8 ml/min after six weeks compared with a lower protein diet [8]. This leads to hyperfiltration and proteinuria in the early stages but eventually results in loss of kidney function [9]. A diet high in protein can cause intraglomerular hypertension, leading to glomerular hyperfiltration, injury, and proteinuria. In a population study of 1522 individuals aged 45 to 64 in Gubbio, it was found that those who consumed more protein had a lower eGFR after 12 years, regardless of whether they had chronic kidney disease (CKD) or not [10]. An additional long-term observational study of 1800 Iranian participants followed over six years showed that a high protein diet also had a high risk of CKD (odds ratio 1.48; 95% CI, 1.03 - 2.15) [11]. 

In cases of living kidney donation, individuals with congenital or acquired solitary kidneys generally have reduced renal mass. As mentioned above, an increased risk of renal hypertrophy is caused by an increased protein diet due to physiological adaptation. This leads to an even higher risk of unfavorable clinical outcomes. It is advised that people with a solitary kidney should avoid a high protein intake of >1.2g/kg/per day. In comparison, individuals with a more alkaline diet have been shown to delay their CKD progression [12].

Some proven advantages to the keto diet have been reported in the literature. The most common clinical use of the keto diet is for refractory epilepsy in children. Hormones that are released and suppressed during fasting and eating are also involved in the initiation and suppression of seizure activities [13]. One study by Jacob et al. also showed the benefit of ketosis in decreasing the cysts in polycystic kidney disease. The study suggested that the activity of the mTOR molecule activated in polycystic kidney disease is highly dependent on the presence of glucose. A reduction in food intake as small as 23% has shown a dramatic decrease in renal cyst growth [14].

At least 11 international studies have studied the relationship between a low-protein diet and renal insufficiency, and eight did not find any benefit of a low-protein diet for preventing renal insufficiency progression [15]. From the studies, Cochrane systemic, which was recently conducted, showed a very low protein diet has a chance of decreasing the number of patients with CKD progression [15].

## Conclusions

Keto diet is one of the most widely accepted weight loss regimens. Few cases exist in the literature regarding the keto diet causing acute kidney injury. Our patient presented with acute kidney injury without an underlying kidney condition. Studies show that individuals with prior kidney disease are at a higher risk of complications. In light of these findings, the ketogenic diet for weight loss should be considered with care, especially in those at high risk of developing kidney disease. 
